# Ginkgolide B Protects Cardiomyocytes from Angiotensin II-Induced Hypertrophy via Regulation of Autophagy through SIRT1-FoxO1

**DOI:** 10.1155/2021/5554569

**Published:** 2021-06-23

**Authors:** Qingyuan Jiang, Ming Lu, Jinyu Li, Zhongsheng Zhu

**Affiliations:** Department of Cardiology, Shanghai Pudong Hospital, Fudan University Pudong Medical Center, Pudong New District, Shanghai 201399, China

## Abstract

Ginkgolide B (GB) is an active ingredient extracted from Ginkgo biloba leaves. However, the effects of GB on cardiac hypertrophy remain unclear. The study is aimed at determining whether GB could alleviate cardiac hypertrophy and exploring its underlying molecular mechanism. Rat cardiomyocyte cell line H9c2 cells were pretreated with GB and incubated with angiotensin II (Ang II) to simulate an in vitro cardiac hypertrophy model. Cell viability, cell size, hypertrophy markers, and autophagy were determined in H9c2 cells after Ang II treatment. Proteins involved in autophagy and the SIRT1 pathway were determined by western blot. Our data demonstrated that GB attenuated Ang II-induced cardiac hypertrophy and reduced the mRNA expressions of hypertrophy marker, atrial natriuretic peptide (ANP), and *β*-myosin heavy chain (*β*-MHC). GB further increased Ang II-induced autophagy in H9c2 cells and modulated expressions of autophagy-related proteins Beclin1 and P62. Modulation of autophagy using autophagy inhibitor 3-methyladenine (3-MA) could abrogate GB-downregulated transcription of NPPA. We then showed that GB attenuated Ang II-induced oxidative stress and reduction in SIRT1 and FoxO1 protein expression. Finally, the effect of GB on autophagy and cardiac hypertrophy could be reversed by SIRT1 inhibitor EX-527. GB inhibits Ang II-induced cardiac hypertrophy by enhancing autophagy via the SIRT1-FoxO1 signaling pathway and might be a potential agent in treating pathological cardiac hypertrophy.

## 1. Introduction

Cardiac hypertrophy is a compensatory response after injury, and its main pathological feathers are enlargement of myocardial cell size and increase of protein synthesis, including physiological hypertrophy and pathological hypertrophy [1]. Physiological hypertrophy is benign, mainly referring to compensatory and adaptive changes of the heart to external stimuli such as physical exercise, without pathological characteristics [2]. Pathological hypertrophy is harmful, causes apoptosis and necrosis of myocardial cells, increases cardiac fibroblasts, and finally makes the functional cardiomyocytes be replaced by fibrous connective tissue [3]. Previous studies have shown that persistent pathological hypertrophy is a key progressive factor and predictive marker of heart failure [4]. Therefore, the prevention of myocardial hypertrophy is important for inhibiting the development of heart failure and improving patients' prognosis.

Angiotensin II (Ang II) is an essential humoral regulator in blood pressure and is a crucial effector of the renin-angiotensin system (RAS). Ang II exists not only in the systemic circulation but also in many organs, such as the brain, blood vessels, kidneys, and heart [5, 6]. In addition, to directly affect hemodynamics, Ang II is also a critical cell growth factor, which can induce cardiomyocyte hypertrophy, promote cardiac fibroblast proliferation, and induce cardiomyocyte apoptosis [7, 8]. Therefore, Ang II is an essential stimulating factor for cardiovascular diseases with hypertrophy, such as hypertension and heart failure [9].

GB is one of the main active ingredients extracted from Ginkgo biloba leaves. It has a wide range of biological and pharmacological effects [10]. GB can protect cardiomyocytes from ischemia-reperfusion-induced injury [11, 12]. It can effectively inhibit doxorubicin-induced cardiomyocyte injury, and its mechanism mainly includes regulating intracellular reactive oxygen species and calcium signal [13]. Recent studies have also shown that another active ingredient from Ginkgo biloba, Ginkgolide A, plays a protective role in myocardial remodeling by improving antioxidant capacity and nitric oxide in pressure overload mice [14]. As GB has a wide range of protective effects on cardiomyocytes, we speculate that GB can inhibit myocardial hypertrophy in cardiomyocytes. However, the protective effect of GB on Ang II-induced myocardial hypertrophy has not been reported.

In this study, we investigated whether GB could attenuate Ang II-induced cardiac hypertrophy in H9c2 cardiomyocytes. Mechanistically, we found that autophagy and the SIRT1-FoxO1 pathway play a crucial role in the protective effects of GB on hypertrophy.

## 2. Methods and Materials

### 2.1. Cell Culture

H9c2 cells were purchased from the American Type Culture Collection (Manassas, VA, USA) and maintained in high-glucose DMEM containing 10% fetal bovine serum (HyClone, USA), penicillin (100 units/mL), and streptomycin (100 mg/mL). Cells were cultured in an incubator with 5% CO_2_ at 37°C. The culture media were replaced every day until the cell density reached 80-90% confluence.

### 2.2. Cell Viability

Cell viability was evaluated using an MTT assay. H9c2 cells (1 × 10^4^ cell/well) were seeded in DMEM in 96-well plates. Cells were incubated with Ginkgolide B of 0, 10, 30, 50, and 100 *μ*M (sc-201037, Santa Cruz, CA, USA) for 48 h. Then, the supernatant was discarded and added to 0.5 mg/mL MTT solution (20 *μ*L/well) for 4 h incubation at 37°C. Then, the medium was added with dimethyl sulfoxide (100 *μ*L/well) to dissolve the formazan crystals. Absorbance was measured at 570 nm using a microplate reader.

### 2.3. Oxidative Stress

Following treatment, H9c2 cells were lysed by RIPA lysis buffer. The malondialdehyde (MDA) content (Cat no. S0131S) and superoxide dismutase (SOD) activity (Cat no. S0109) were determined using commercial assay kits (Beyotime, Nantong, China). The MDA was expressed as nmol/mg protein, and SOD was defined as U/mg protein.

### 2.4. Immunofluorescence Staining

H9c2 cells were fixed with 4% paraformaldehyde in PBS for 20 min, permeabilized with 0.5% Triton X-100 in PBS for 40 min, and blocked with 5% BSA for 1 h. Then, the cells were incubated with primary antibodies against *α*-actinin or LC3-II (1 : 100 dilutions; ab192890, Abcam, UK) overnight at 4°C, followed by incubation with Alexa Fluor 488-linked secondary antibody (Invitrogen, USA). Finally, nuclei were then counterstained with DAPI (1 : 1000, Sigma-Aldrich). The analysis was performed by Image-Pro Plus 6.0 software to calculate the number of cells with LC3-II+.

### 2.5. Real-Time PCR

Total RNA was isolated using a TRIzol reagent (Invitrogen) and reversely transcribed to cDNA using the PrimeScript RT Reagent Kit (TaKaRa). Sequences for primers are as follows: NPPA (encoding for ANP) forward: 5′-ACC AAG GGC TTC TTC CTC T-3′, NPPA reverse: 5′-TTC TAC CGG CAT CTT CTC C-3′; *β*-MHC forward: 5′-TCT GGA CAG CTC CCC ATT CT-3′, *β*-MHC reverse: 5′-CAA GGC TAA CCT GGA GAA GAT G-3′; and GAPDH forward: 5′-AAC TTT GGC ATT GTG GAA GG-3′, GAPDH reverse: 5′-ACA CAT TGG GGG TAG GAA CA-3′. Real-time PCR was performed with SYBR® Fast qPCR Mix (TaKaRa) using an ABI Prism 7500 Fast Real-time PCR instrument (Applied Biosystems; Foster City, CA, USA). Relative ANP and *β*-MHC mRNA was determined by the 2^-*ΔΔ*Ct^ method. This experiment was repeated three times.

### 2.6. Western Blotting

H9c2 cell lysis was performed in RIPA lysis buffer, and protein was quantified using the BCA assay kit. Protein (50 *μ*g) was separated by 12% SDS-PAGE and transferred onto PVDF membranes. The membrane was blocked with 5% low-fat milk and was incubated overnight at 4°C with primary antibodies against Beclin1 (1 : 200, sc-48341, Santa Cruz), P62 (1 : 200, sc-28359, Santa Cruz), SIRT1 (1 : 100, ab189494, Abcam), FoxO1 (1 : 100, ab52857, Abcam), or *β*-actin (1 : 1000, ab8226, Abcam). The membranes were then incubated with HRP-conjugated secondary antibodies (1 : 5000, Santa Cruz, USA) for 1 h. Bands were visualized using enhanced chemiluminescence (Roche Diagnostics, Basel, Switzerland). Protein expression was quantified using ImageJ software and normalized to that of *β*-actin.

### 2.7. Statistical Analysis

Data from at least three independent experiments were expressed with mean ± standard deviation (SD). All statistical analyses were performed by SPSS 20.0 software. Differences between groups were analyzed by one-way ANOVA, followed by Bonferroni's test. Differences were considered statistically significant when *P* < 0.05.

## 3. Results

### 3.1. GB Protects against Ang II-Induced Cardiomyocyte Hypertrophy

**Figure 1:**
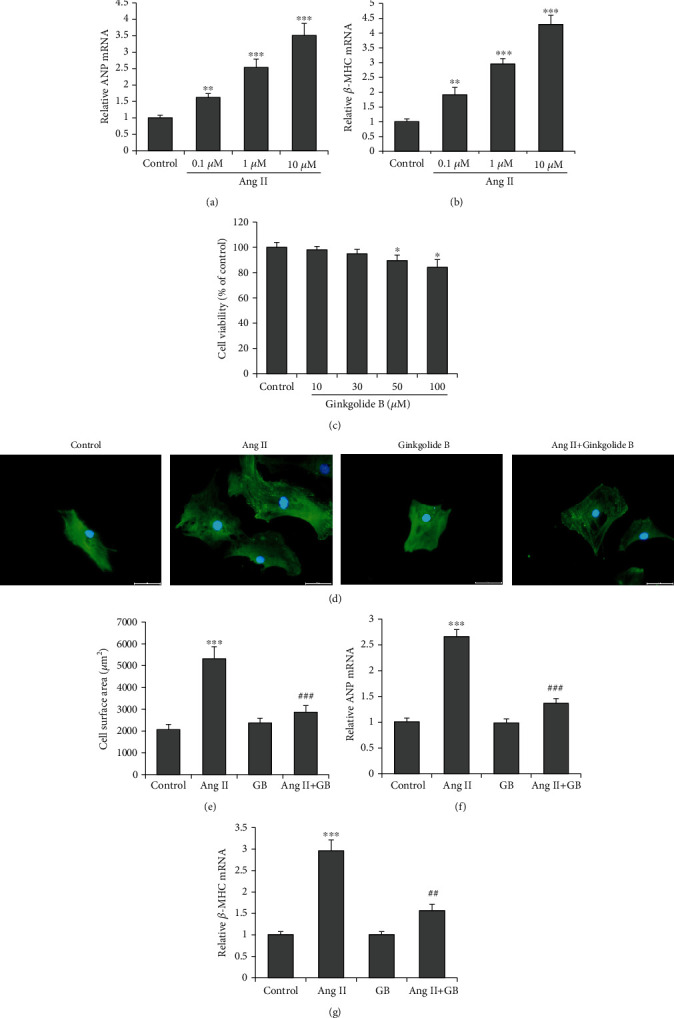
Ginkgolide B attenuates Ang II-induced hypertrophy. H9c2 cells were incubated with Ang II (0, 0.1, 1, and 10 *μ*M) for 48 h. RT-qPCR examined (a) ANP and (b) *β*-MHC mRNA levels. (c) Cell viability assay (cells were incubated for 48 h) showed the Ginkgolide B with above 50 *μ*M has an obvious toxic effect on cardiomyocytes. (d) H9c2 cells were pretreated with Ginkgolide B (30 *μ*M) for 2 h and then incubated with Ang II (1 *μ*M) for a further 48 h. Representative photographs are shown (scale bar: 5 *μ*m). (e) Cardiomyocyte hypertrophy is evaluated by quantifying cell surface area. mRNA expressions of ANP (f) and *β*-MHC (g) were evaluated by real-time PCR. Data were presented as the mean ± SD. ^∗^*P* < 0.05, ^∗∗∗^*P* < 0.001 vs. the control group; ^#^*P* < 0.05, ^##^*P* < 0.01, and ^###^*P* < 0.001 vs. the Ang II group.

To determine GB's effects on cardiac hypertrophy, we used Ang II to induce cardiomyocyte hypertrophy. H9c2 cells were incubated with Ang II at various concentrations for 48 h, and the hypertrophy markers were detected. Ang II increased the mRNA levels of ANP and *β*-MHC in a concentration-dependent manner (Figures 1(a) and 1(b)). H9c2 cells were incubated with GB, and cell viability assay showed an apparent toxic effect on cardiomyocytes above 50 *μ*M (Figure 1(c)). According to the MTT experiment results, Ang II concentration of 1 *μ*M and GB concentrations of 30 *μ*M were selected as the final drug concentration of H9c2 in rat cardiomyocytes. H9c2 cells were pretreated with GB for 2 h and then incubated with Ang II for a further 48 h. Immunofluorescence was performed using an antibody of *α*-actinin. It showed that Ang II markedly increased cardiomyocyte size (cell surface area) of H9c2 cells, and this change was suppressed by GB (Figures 1(d) and 1(e)). The mRNA levels of NPPA and *β*-MHC were increased in Ang II-treated cardiomyocytes. However, GB markedly reversed the Ang II-induced hypertrophic responses (Figures 1(f) and 1(g)). These findings indicate that GB inhibits Ang II-induced cardiomyocyte hypertrophy.

### 3.2. GB Promoted Autophagy in Cardiomyocyte

**Figure 2:**
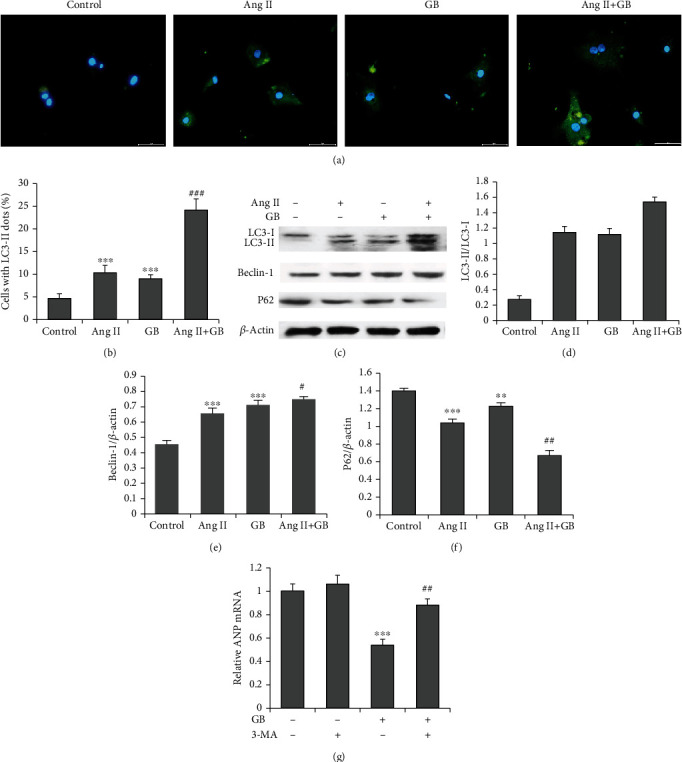
Effect of Ginkgolide B on autophagy in Ang II-induced cardiomyocytes. (a) Fluorescence microscopy of H9c2 cells staining with an LC3-II antibody (green, magnification ×100). (b) Quantification of cells with LC3-II dots. (c) Representative blots of autophagy-related proteins. GB further increases the ratio of LC3-II/LC3-I (d) and Beclin-1 protein expression (e) and decreases the P62 protein (f) in H9c2 cells with Ang II. Cells were pretreated with the autophagy inhibitor, 3-MA (1 *μ*M). ^∗∗^*P* < 0.01, ^∗∗∗^*P* < 0.001 vs. the control group; ^#^*P* < 0.05, ^##^*P* < 0.01, and ^###^*P* < 0.001 vs. the Ang II group. (g) 3-MA significantly attenuates Ginkgolide B-induced inhibition in ANP mRNA expression. ^∗∗∗^*P* < 0.001 vs. the GB group; ^##^*P* < 0.01 vs. the 3-MA group.

To investigate whether GB regulates the autophagy process in Ang II-treated cardiomyocytes, we detected autophagosomes by staining H9c2 cells with LC3-II antibody. The cells were stained with FITC (green puncta) and LC3-II (blue puncta) and might appear several yellow puncta indicators of autophagosomes. The results showed that Ang II-induced autophagy in H9c2 cells, as evidenced by slightly increased yellow fluorescence and GB pretreatment, could further increase yellow fluorescence in H9c2 cells stimulated with Ang II (Figure 2(a)). Quantification analysis showed that GB significantly increased the percentage of cells with LC3-II dots (Figure 2(b)). We then explored the effect of GB on autophagy-related proteins using western blot. The results showed that GB prominently enhanced Beclin-1 and reduced P62 protein expression in H9c2 cells stimulated with Ang II (Figures 2(c)–2(e)). To further investigate the role of autophagy in the protective effect of GB on cardiomyocyte hypertrophy, H9c2 cells were preincubated with an autophagy inhibitor, 3-methyladenine (3-MA). Our results showed that 3-MA abrogated the GB-induced reduction in the mRNA expression of NPPA (Figure 2(f)). These data suggest that GB protects against Ang II-induced cardiomyocyte hypertrophy by promoting autophagy.

### 3.3. GB Suppressed Oxidative Stress and Activated SIRT1-FoxO1 Pathway in Cardiomyocyte with Ang II

**Figure 3:**
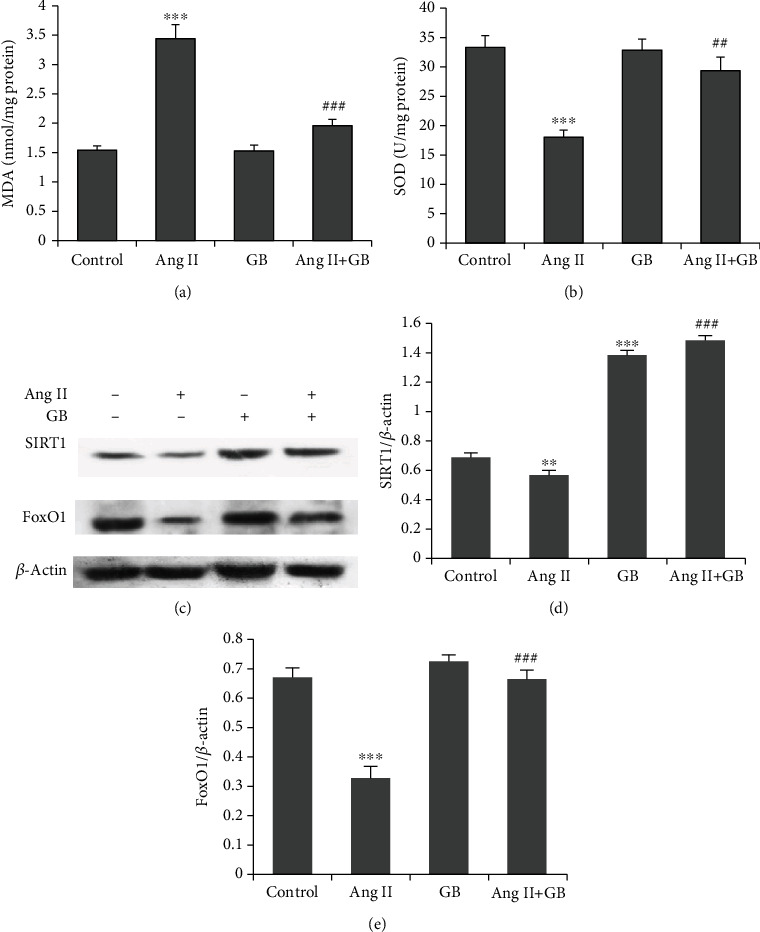
Effect of Ginkgolide B on oxidative stress and the SIRT1-FoxO1 pathway in Ang II-induced cardiomyocytes. Oxidative stress markers are measured, including MDA content (a) and SOD activity (b) in H9c2 cells. (c) Representative blots of protein levels of SIRT1 (d) and FoxO1 (e) are shown. ^∗∗^*P* < 0.01, ^∗∗∗^*P* < 0.001 vs. the control group; ^##^*P* < 0.01, ^###^*P* < 0.001 vs. the Ang II group.

MDA and SOD are important biomarkers of oxidative stress and are widely used as indicators of oxidative injury. To examine the effect of GB on Ang II-induced oxidative stress, the level of MDA and the activity of SOD were measured. The results demonstrated that Ang II clearly increased MDA content and decreased SOD activity in H9c2 cells compared with control cells. However, pretreatment with GB potently decreased the MDA content and restored the SOD activity in Ang II-stimulated H9c2 cells (Figures 3(a) and 3(b)). We next performed western blot analysis to measure the protein expression of SIRT1 and FoxO1 (Figure 3(c)). The results showed that Ang II significantly reduced the expression of SIRT1 and FoxO1 in H9c2 cells, which could be reversed by GB (Figures 3(d) and 3(e)). These results reveal that GB inhibits Ang II-induced oxidative stress and inactivation of the SIRT1-FoxO1 signaling pathway.

### 3.4. SIRT1 And FoxO1 Mediate Suppressive Effect of GB on Cardiomyocyte Hypertrophy

**Figure 4:**
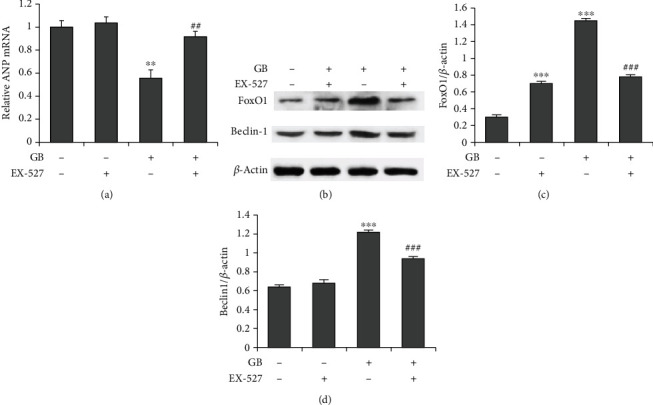
SIRT1 mediates the protective effects of Ginkgolide B on Ang II-induced hypertrophy in cardiomyocytes. H9c2 cells were pretreated with Ginkgolide B (30 *μ*M) and SIRT1 inhibitor, EX-527 (100 nM), for 2 h and then incubated with Ang II (1 *μ*M) for further 48 h. (a) ANP mRNA level after SIRT1 inhibition. (b) Representative blots of SIRT1 and autophagy-related proteins. EX-527 abolishes Ginkgolide B's promotive effect on (c) FoxO1 and (d) Beclin-1 protein expressions. ^∗∗^*P* < 0.01, ^∗∗∗^*P* < 0.001 vs. the GB group; ^###^*P* ≤ 0.001 vs. the EX-527 group.

To investigate the role of SIRT1 in the protective effect of GB on cardiomyocyte hypertrophy, H9c2 cells were stimulated with Ang II and different combinations of GB and SIRT1 inhibitor, EX-527. The results showed that cotreatment with EX-527 could abrogate the reduction in NPPA mRNA expression induced by GB compared with those stimulated with GB alone (Figure 4(a)). We then investigated the role of SIRT1 in its downstream pathway and autophagy-related proteins using western blot. Our results showed that cotreatment with EX-527 also reversed the GB-induced increase in the expression levels of FoxO1 (Figures 4(b) and 4(c)) and Beclin-1 (Figures 4(b) and 4(d)). Our findings suggested that GB promotes autophagy and inhibits Ang II-induced cardiomyocyte hypertrophy through the SIRT1-FoxO1 signaling pathway.

## 4. Discussion

In the present study, we investigated GB's role and mechanism in Ang II-induced cardiac hypertrophy. We demonstrated that GB markedly suppressed cardiac hypertrophy, autophagy, and oxidative stress and activated the SIRT1-FoxO1 pathway in H9c2 cells. Our experiments show that GB's potential effect on hypertrophy and autophagy is partially mediated by the SIRT1-FoxO1-pathway in Ang II-induced cardiomyocytes.

The results showed that GB had a slightly suppressive effect on cell viability of cardiomyocytes at high concentrations, while GB at lower concentrations demonstrated a cardioprotective effect. Some embryonic genes have been activated in the adult heart with hypertrophy [15]. In this study, RT-qPCR was applied to determine these hypertrophy-related genes. Ang II treatment for 48 h significantly upregulated the mRNA levels of NPPA and *β*-MHC, but these changes were markedly reversed after treatment with GB. The results suggest that GB can effectively inhibit the expression of a hypertrophic gene induced by Ang II and play a role in slowing down hypertrophy process. *α*-Actinin is the main component of myofibrillar Z-disk, which may play a role in the stress and transmission of myocardial cells. When cardiac hypertrophy occurs, the traction and contraction force of myofibrils are enhanced, and the expression of *α*-actinin will increase with the occurrence of myocardial hypertrophy [16]. In this study, the expression of *α*-actinin was significantly increased under the induction of Ang II, but GB and Ang II could inhibit the expression of *α*-actinin. GB has shown cardioprotective properties in cardiomyocytes induced by ischemia-reperfusion and doxorubicin [11–13]. This study adds cardiac hypertrophy as another cardioprotective function for GB. There are complex interactions between cardiac hypertrophy and myocardial ischemia-reperfusion (IR) injury. After IR injury, cardiomyocytes have tissue repair processes, including hypertrophic remodeling [17]. Conversely, the heart of patients with hypertension and cardiac hypertrophy has a higher sensitivity to IR injury [18]. Therefore, it remains unknown whether some common mechanisms are underlying the cardioprotective effect of GB against cardiac hypertrophy and IR injury.

Our results show that Ang II induced autophagy in H9c2 cells. Meanwhile, GB further enhanced Ang II-induced autophagy, as evidenced by markedly higher fluorescence intensity of LC3-II in cells with GB and Ang II compared to cells with Ang II alone. Autophagy is a cellular process for the degradation of damaged cytoplasmic contents for catabolism and recycling and plays a critical pathogenic role in cardiac hypertrophy. However, there are conflicting reports on the role of autophagy in Ang II-induced cardiac hypertrophy [19]. Some studies showed that inhibition of the Ang II-induced autophagy suppressed cardiac hypertrophy [20, 21]. In contrast, others showed that activation of Ang II-induced autophagy suppressed cardiac hypertrophy [22, 23]. Our study indicates that GB further enhanced Ang II-induced autophagy and suppressed cardiac hypertrophy, consistent with the previous report that induction of autophagy prevents cardiac hypertrophy [24]. The results suggest that autophagy insufficiency results from Ang II. Pressure overload is a contributing factor to cardiac remodeling and hypertrophy [25] as GB, and its analog GB enhanced autophagy in cancer cells and astrocytes [26, 27]. GB might be a promising cardioprotective agent in Ang II-stimulated cardiac hypertrophy through enhancing autophagy.

This study shows GB suppressed oxidative stress of cardiomyocytes, as evidenced by decreased MDA content and increased SOD activity in Ang II-stimulated H9c2 cells. Oxidative stress is an active cellular response to increased reactive oxygen species (ROS), which are active oxygen-containing compounds generated during aerobic metabolism. Oxidative stress is a potent inducer and promotor of cardiac hypertrophy through various cellular signaling pathways, especially the extracellular matrix (ECM) [28, 29]. GB can protect against endothelial dysfunction through inhibiting oxidative stress in diabetic rats, as evidenced by reduced NO bioavailability and SOD activity and increased MDA content in aortic tissues [30]. Moreover, in addition to the inhibition of oxidative stress, GB also alleviated cardiac fibrosis in diabetic rats by decreasing the expression of TGF-*β*1 [31], a protein closely associated with the hypertrophic signaling pathway [32]. However, the signaling pathways underlying Ang II-induced oxidative stress remain unclear.

In this study, Ang II treatment reduced SIRT1 and FoxO1 expressions in cardiomyocytes, which was reversed by GB. SIRT1 is a class III histone deacetylase modulates tissue homeostasis by deacetylating downstream target proteins [33]. Of note, activation of SIRT1 by GB has been observed in endothelial cells and ischemic stroke [34, 35]. Previous studies showed that SIRT1 activation could reduce Ang II-induced cardiac hypertrophy [36, 37]. SIRT1 has also been shown to regulate autophagy through FoxO1, thus improved adaptive cardiac remodeling [38]. Furthermore, SIRT1 increased starvation-induced autophagy through deacetylation of FoxO1, which in turn maintained hemostasis of cardiomyocytes during starvation [39]. Our study shows that SIRT1-FoxO1 is the target pathway proteins activated by GB in Ang II-stimulated cardiomyocytes.

## 5. Conclusions

GB can effectively inhibit cardiac hypertrophy in vitro. More importantly, SIRT1-FoxO1-mediated autophagy is the primary mechanism underlying GB's cardioprotective effect in cardiac hypertrophy. GB is a promising agent for combating cardiac hypertrophy, and more detailed mechanisms should be investigated in vivo experimental model.

## Data Availability

The data in support of the results are available from the corresponding author on reasonable request.

## Abbreviations

Ang II: Angiotensin II
ANP: Atrial natriuretic peptide
*β*-MHC: *β*-Myosin heavy chain
GB: Ginkgolide B
MDA: Malondialdehyde
RAS: Renin angiotensin system
SOD: Superoxide dismutase.
